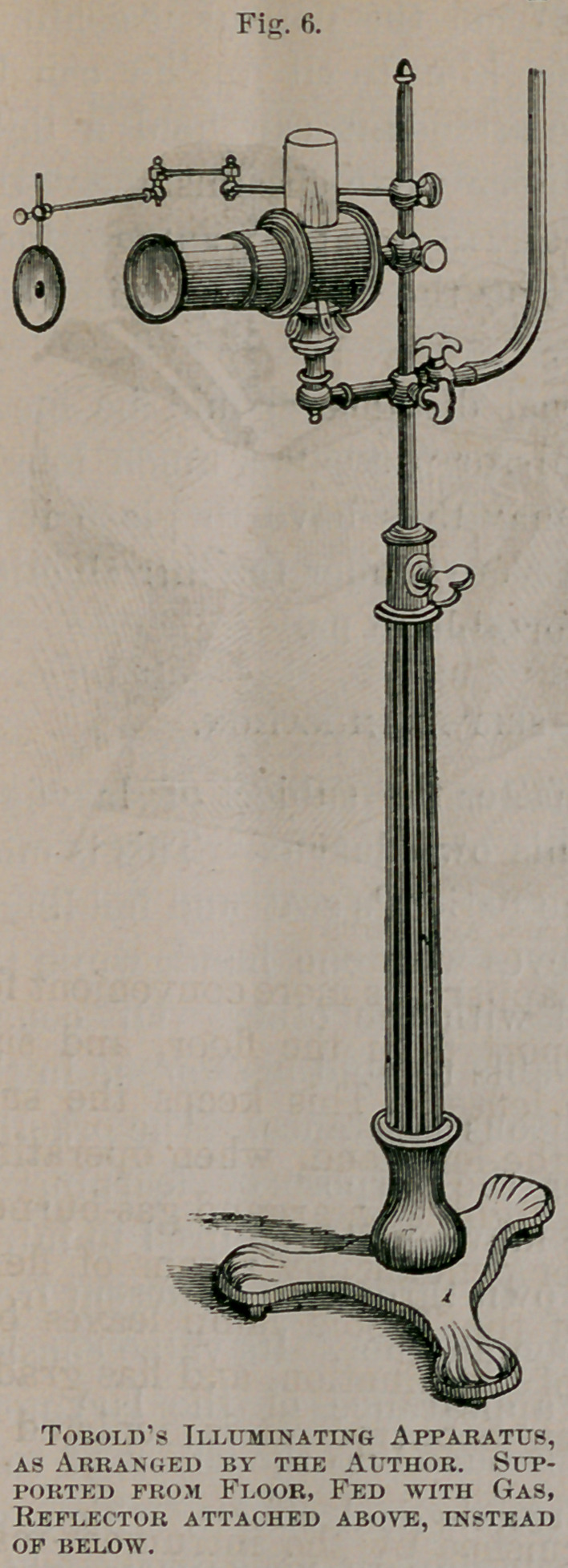# Brief Exposition of the Management of the Laryngoscope

**Published:** 1871-10

**Authors:** J. Solis Cohen

**Affiliations:** Lecturer on Laryngoscopy, and Diseases of the Throat and Chest, in Jefferson Medical College


ATLANTA

MEDICAL AND SURGICAL JOURNAL.

NEW SERIES.

VOL. IXB ATLANTA, GEORGIA, OCTOBER, 1871. NO. 7.

### A BRIEF EXPOSITION OF THE MANAGEMENT OF THE LARYNGOSCOPE.

BY J. SOLIS COHEN, M.D.,

Lecturer on Laryngoscopy, and Diseases of the Throat and Chest, in Jefferson Medical College.

The following pages will convey, in as concise a manner as the author can command, the essential instruction requisite for the management of the laryngeal mirror in the diagnosis of affections of the throat.

The laryngeal, or throat mirror, consists of a small section of looking-glass securely attached to a handle, as represented in figure 1, (see page 368.) It is constructed for the purpose of being passed through the mouth into the pharynx, and being held for a few moments in such a position that it will reflect to the eye of the observer an image of the interior of the larynx, and of other structures adjacent. It thus permits the inspection of parts beyond the limits of direct visual examination, and is, consequently, of immense value to the medical practitioner.

A good light is an essential pre-requisite to a laryngoscopic examination; and when this is furnished by the solar rays the

Page(s) missing

Fig. 1.

THE LARYNGEAL MIRROR.

only instrument needed for the examination is the simple mirror. When the sun-light is insufficient for this purpose, we resort to certain accessory apparatus to concentrate its rays; otherwise, we make use of artificial illumination.

The form of the laryngeal mirror is unimportant. The most convenient form is that depicted in the text; and it will meet almost every indication. It is a circular glass mirror, one inch in diameter, mounted in German silver, and soldered at an angle of 120°, to a stout German silver shank, which, with the wooden handle, is eight inches long. It sometimes becomes necessary to use a smaller mirror, and, occasionally, we can employ one much larger. When the tonsils are very much hypertrophied we use an oval mirror, one inch in its long diameter, and from five-eighths to seven eighths of an inch transversely, according to circumstances.

The position of the mirror in the pharynx, and the manner of using it, is depicted in figure 2, (see page 369), which shews the image reflected from its surface.

The mirror is represented at an angle of about 45° with the plane of the larynx; but, in practice, its position will vary in different individuals, in consequence of peculiarities of structure.—Much, too, will depend upon the degree of retroflexion given to the patient's head, the position of the observer's eye, and other circumstances which will become apparent as we proceed in the discussion of our subject.

The manner in which the laryngeal mirror is most conveniently used is as follows:

The patient is seated in such position that a strong light shall illumine the pharynx, the lower portion of the soft palate especially. The examination may be made in the open air, or before a window. The observer sits in front of his patient, at such distance as to obtain distinct and clear vision of the soft palate and of the posterior wall of the pharynx. The head of the patient should be held erect, or be very slightly bent backwards; and the position may have to be varied from

Fig. 2.

THE LARYNGEAL MIRROR IN POSITION.

the one to the other after the mirror has been introduced ; but for the majority of cases, a favorable position will be such a one as shall place the lower border of the upper incisor teeth upon a plane horizontal with the base of the soft palate. The mouth should be widely distended, and the tongue thrust forwards and downwards with considerable muscular force, its body lying upon the floor of the mouth, and its posterior portion and base rendered as concave as possible. In this position, its protruding tip may be enveloped in the fold of a handkerchief or napkin, and be held by the patient himself, or by the observer if he prefer it—the napkin being interposed to prevent the tongue from slipping back from between the thumb and fingers. The patient should breathe quietly, but rather deeply, and without effort or panting.

The stem of the mirror should be taken in hand just as one would handle a pen; the wrist being well extended, though not stiffly so, with the mirror pointing upwards, and its reflecting surface horizontal and looking downwards, as shewn in figure 3.

MANNER OF HOLDING THE MIRROR WHEN ABOUT INTRODUCING IT.

The patient being told to take a deep respiration, so as to raise the soft palate, the laryngeal mirror is passed well above the tongue, directly backwards, until it reaches the uvula; when, receiving the uvula on the back of the mirror, the wrist is flexed, and the mirror landed with its lower border on the posterior wall of the pharyx, the uvula and soft palate being pushed backwards and somewhat upwards in the manœuvre. The stem of the mirror will now be horizontal. and the reflecting surface present obliquely downwards and forwards. When the palate is raised very high, during a deep inspiration, we can place the mirror in position without first touching the palate, which is then allowed to fall upon the back of the mirror as the act of expiration is effected. This procedure will be found serviceable in the examination of nervous individuals.

To prevent the halitus of the breath from depositing on the mirror, its reflecting surface is held, for a moment or two, over a flame until the instrument has acquired a heat which is not disagreeable to contact with the hand or cheek. This point will be reached, if the mirror be perfectly cold, as soon as the condensed moisture passes off from circumference to center, as the mirror is held above the flame. The mirror may also be warmed by immersion in hot water. Care must be taken not to burn the patient with too hot a mirror.

The mirror being properly introduced, we perceive in it an image of the larynx and adjacent structures, but in a reversed position, though not an inverted one; that is to say, those structures, which are posterior in the body, are anterior in the image, and what is really in front looks as though it were behind, the relative positions of right and left being unchanged. A little reflexion will, with the aid of the figure 4, render the conception of the image intelligible.

Fig. 4.

Relative Position of the Larynx, and its Reflection in the Mirror Above.

The structures (base of tongue and epiglottis), which are in front in the patient, appear above and behind in the mirror; the parts (arytenoid cartilages, etc.,) which are below and behind in the patient, appear below and in front in the mirror; but the structures, which, in the patient, are in reality on the right hand of the observer, are on his right in the mirror also. In other words, those parts nearest the mirror are seen as if they were nearer the observer, who sees them much as he would do could he look at them from behind, with his eye in the position occupied by the laryngeal mirror. The lower cut in the figure represents a view of the base of the tongue and the larynx, in the relative positions they bear in the party under examination; while the upper cut shows the image as seen in the mirror. If the reader will hold a laryngeal mirror (or, in lieu of it, a piece of looking-glass) obliquely above the lower figure and behind it, so as to receive its reflection, he will see an image such as is pictured in the upper figure. This diagram will be found useful in studying the relations of parts in actual practice.

The mirror must not be retained in the mouth too long at a time. It is better to re-introduce it several times than to fatigue the parts by keeping them too long in an uneasy position. In this way we avoid the induction of congestion, or of irritability and spasm.

### IMPEDIMENTS TO THE EXAMINATION.

As a rule, an experienced laryngoscopist experiences no difficulty whatever in effecting a satisfactory examination at the very first introduction of the mirror.

Occasionally, however, impediments are presented, of which some consideration is necessary.

There may be an unwillingness to open the mouth properly, or even an inability to do so; and it is very necessary that the mouth should be opened widely; the more widely the better. Some patients open the mouth well enough, but close it involuntarily as soon as the attempt is made to introduce the instrument. If a little moral suasion is insufficient to counteract this propensity, we resort to a mouth distender, or a speculum, and pass the laryngeal mirror through it. Of these there are many forms. A short glass speculum, similar to that used in vaginal exploration, but unsilvered and not blackened, about an inch and an eighth in diameter, will answer the purpose in most instances. Under these circumstances the tongue is retained in the mouth, and is kept depressed by the position of the speculum. This speculum may be further utilized in making applications to the throats of refractory patients.

The management of the tongue sometimes becomes a matter of considerable annoyance, rising up as soon as the instrument passes the teeth, and, sometimes, pushing the mirror to the very roof of the mouth. It is necessary that the tongue should be directed forwards and downwards, so as to increase the space in the pharynx, and to draw the epiglottis up by the tension upon the glotto-epiglottic ligament; for, in most people, the laryngeal entrance is overlooked by the epiglottis, which, unless it is moderately erect, will, to a greater or lesser extent, intercept the view of the intra-laryngeal structures. It will be found a good plan to instruct the patient to hollow his tongue at the base, and then thrust it forcibly out of the mouth, when, if he cannot retain it in position himself, it may be held by the thum[illegible] and fingers of the disengaged hand of the observer. If there is any risk of injury to the frenum, a compress may be placed over the lower incisor teeth. Much ingenuity has been manifested in the invention of depressors and forceps for retaining the tongue in position; but the employment of a mechanical contrivance for this purpose is greatly to be deprecated, and should be avoided, as a rule. Occasionally, it does seem impossible to get along without something of the kind; but the alternative is to be acknowledged with reluctance. If the tongue is so fleshy as to occupy too much space for our purpose in the cavity of the mouth, or so restless that it keeps bobbing about, we can often press it down, or restrain its movements, by the simple contact of a pen-handle, probe, or even the fore-finger; something to steady it, as it were.

Irritability of the fauces is another great obstacle occasionally presented, though by no means as frequently as general opinion presumes. Nearly every unsuccessful attempt at laryngoscopic examination attributed to irritability of the patient's fauces, is due to irritability in the hand of the manipulator. Want of skill on the part of the examiner, and want of patience also, once overcome, irritability of the fauces is rarely encountered. Sometimes, however, there does exist a great deal of irritability of the fauces; but the cases are very few, and far between. It may often be overcome by moral suasion. Gentle manipulation of the parts with a foreign body will often succeed. Astringent or narcotic solutions may be applied locally, in spray, or by the mop. When there is time to await its action, and no contra-indication to its employment, the administration of large doses (grs. 30—60) of bromide of potassium, at intervals of three or four hours, for three or four successive doses, will sometimes induce a tolerance of manipulation. Gargles and sprays of alum, tannin, the bromides, sulphuric ether, rhigolene, and chimogene; pencillings with astringents and caustics, with solutions of morphia and chloroform; the local contact of ice; the inhalation of a few drops of chloroform, and a still longer list of other plans have been recommended, many of the most inefficient of which have been highly extolled, possibly because they overcame the irritability in the only case in which they were tried. Of all these plans, the best are the contact of the spray of a nebulized solution of tannin, and the inhalation of a few whiffs of chloroform; but a still more judicious plan, without any drawbacks, is that of overcoming the sensibility of the parts by repeated contact of the laryngeal mirror. This irritability is most apt to exist during the digestion of food, and may often be met successfully by waiting until the stomach has discharged its contents into the bowel. When marked disorder of the digestive apparatus exists, a smart purge, administered at night, will lessen the sensitiveness of the fauces on the following morning.

Enlargement of the tonsils often renders the employment of oval mirrors necessary. If the mirror is broader than the space between the hypertrophied glands, it should be pushed right between them and behind, and, although they may cover the sides of the mirror, there will usually remain sufficient reflecting surface exposed to ensure a satisfactory examination. The tonsils must be passed with great celerity, and the act is then hardly recognized by the patient. If the tonsils are hypertrophied to such an extent as to preclude the introduction of an oval mirror, they must be excised.

Elongation of the uvula may become a source of difficulty by hanging below the mirror, so that its own reflection intercepts the view of the parts to be examined. If it cannot be retracted by tittillation or astringent applications, its exuberant portion must be clipped off.

An unfavorable position of the epiglottis is an obstacle much more serious than any yet considered. Here, nature has placed an impediment to the examination of the larynx. Sometimes as a congenital conformation, sometimes as the result of cicatrization, sometimes as an acquisition dependant upon a vicious mode of utterance in public speaking, we meet occasionally a depressed epiglottis, overhanging the vestibule of the larynx to such an extent as to preclude the passage of light to its interior. When this condition exists in but slight extent, and especially in acquired cases, it may be overcome by frequent pulling of the valve forward with the finger, which the patient can very readily be instructed to do for himself. A momentary view of the larynx can often be obtained, in cases of this kind, by causing the patient to make an ironical laugh at the moment of examination, or a vocal sound during inspiration, or a sudden inspiration, or to make the sound of *e*, as in beg, in a high-pitched tone. These movements throw the epiglottis upward for the moment. Where immediate examination is necessary, we can resort to a bent probe of stout wire, whalebone, or hard rubber, which is passed over the laryngeal face of the epiglottis, upon which traction forward is gently but firmly exercised. Forceps and needles of various kinds have been employed for this purpose, but they inflict injury on the epiglottis, and are otherwise unnecessary. A good instrument for managing a depressed epiglottis has yet to be devised. Where cicatricial tissue is the cause of depression, the knife becomes requisite.

The manner of breathing sometimes offers an impediment to the examination. Nervous individuals are sometimes excited by the paraphanalia incident to an examination, and are apt to breathe in a hurried, constrained or spasmodic manner. This irregular respiration must be overcome, preparatory to a successful result. By beating time for inspiration and expiration, by inducing the patient to follow one's own respirations, or some analagous device, we readily succeed in restoring the patient's confidence, and then proceed to the examination as quietly and as gently as may be.

The great element of success in laryngoscopic manipulation, is patience. It is useless to hurry a patient, or to scold him; this but excites him the more, and the greater his excitement or dread of the manipulation, the greater will be his susceptibility to spasm from contact of the mirror. If time enough to proceed deliberately cannot be devoted to the object, it had better be abandoned, or postponed to a more convenient period.

Anesthesia is inapplicable to the requirements of laryngoscopy, because the co-operation of the patient is necessary for the production of physiological movements which shall bring into view the various parts of the structures under examination.

### APPARATUS TO INCREASE THE ILLUMINATION.

The manipulation of the laryngeal mirror is substantially the same whatever may be the source of illumination. Direct sunlight is available only during that period of the day when the sun's rays incline to the horizontal. When the time of day, or location of room, is unfavorable to the utilization of the direct rays from the sun, we may reflect the light to the desired point by means of a small looking-glass. A cone of light may be thus reflected to a distant point of the apartment, say against a wall, and the patient be then placed so that his mouth will intercept the cone. The pharynx will then be brilliantly illuminated, and the examination can be proceeded with, as already detailed. As the day grows older, the position of the patient's chair will have to be altered in compliance with the track of the sun. Sometimes a plane mirror, attached to the forehead of the observer, is used as a reflector of direct solar light.

More frequently, and more conveniently, a concave mirror is used to reflect the diffuse daylight of the apartment. This is the laryngoscopic reflector devised by Czermak. It consists of a concave mirror of circular outline, about three and a half inches in diameter, with a focus suited to the visual distance of the observer. One with a focus of from eight to twelve inches, can be used by most persons. The patient is placed with the light to his back, or to one side; the observer is directly opposite, with the reflector in one hand, or upon a stand at his side, or attached in some convenient manner to his forehead; the reflector being so mounted as to be susceptible of inclination in every direction. The light is then received upon the reflector, and thence reflected into the mouth, upon the spot to be occupied by the laryngeal mirror.

### EXAMINATION BY ARTIFICIAL LIGHT.

In employing artificial illumination, we may use either direct or reflected light; the former method being the favorite one pursued in France, and the latter that generally preferred in Germany, Great Britain, and the United States. The best light for the purpose, is gas or coal oil. Coal oil furnishes the whiter and more constant light; gas in the more convenient in management.

In order to increase the power of the light, it is customary to place a condensing lens in front of it. In examination by direct light, the lamp, with the lens in front of it, is placed upon a small stand or table, behind which the observer sits; a shade behind the light protects his eyes from its glare. The patient is seated in front of the table, with his face to the light, which is placed at such a height that its rays may pass directly into his mouth. The examiner passes his arm around the lamp on one side and makes the exploration, as by solar light. This is a very good method, requiring but little apparatus; but it is rather awkward, in consequence of the position of the light, which is between the patient and observer. The direction of the light cannot be altered, without suspending the examination. It is far inferior in convenience to examination by by reflected light, though perhaps occasionally more advantageous in affording a brighter illumination.

In examining by reflected light, we place the lamp most conveniently to one side of the patient, usually the right side, a little behind his head and about the level of his ear; or we may place the light directly behind the patient, and above his head. Then, sitting in front of him, we receive the rays of light upon a concave reflector, having one-half the focal distance of that with which we work by sunlight. Under these circumstances, we use the disc of light just within, or just beyond the inverted image of the flame, as the illuminating medium, and it affords but a small amount of light. With a condensing lens in front of the light, we increase its power and are enabled to cast a large circle of illumination into the mouth.

The best illuminating apparatus yet devised for laryngoscopic examination, is that of Tobold, depicted in figure 5.

The source of illumination here represented is a coal oil lamp capable of being set at any elevation upon the support, and of being changed at will. This is surrounded by a tube containing a series of three condensing lenses for increasing the light. The reflector is attached to the illuminating apparatus by a jointed, movable arm. In using this apparatus, the flame should be placed about on a level with the patient's mouth.

Fig. 5.

TOBOLD'S ILLUMINATING APPARATUS.

The author has rendered this apparatus more convenient for constant use, by taking the support from the floor, and suspending the reflector above the lenses. This keeps the supporting arm out of the way of the left hand, when operating upon the larynx. The source of light is an argand gas-burner, fed from a convenient bracket or pendant, by means of flexible tubing. This adaptation of the Tobold lamp leaves but little to be desired in the way of illumination, and has gradually come into general use in this country. It is depicted in figure 6.

Nearly all the reflectors furnished by the intrument-makers, are perforated in the centre. This is due to the fact that Czermak took the idea of the laryngoscopic reflector from that of the ophthalmoscope. The perforation is by no means essential, though it may be occasionally used with advantage, so as to look in the very axis of the rays of light. The re flectors attached to the head are sometimes suspended before one eye. In that case, they must be perforated. A band, a pad and spring passing over the top of the head, or a spectacle frame, are the usual means of attaching the reflector to the head. The most convenient method, perhaps, is to make use of a band of elastic webbing encircling the head.

Fig. 6.

Tobold's Illuminating Apparatus, as Arranged by the Author. Supported from Floor, Fed with Gas, Reflector attached above, instead of below.

In employing artificial light, we must shut out any excess of daylight. A dark shade before the window suffices. It is unnecessary to exclude the sunlight to such an extent as to render it difficult to distinguish the various objects in the room.

The reflector attached to the head is very serviceable in examining patients who are confined to bed; but under all other circumstances, a steady support will be found to yield much more satisfactory results. Still, the use of the head reflector is preferred by a good many, and some of the most valuable triumphs in laryngeal surgery have been accomplished by its aid, and this even without the use of any condensing apparatus to increase the light given by an ordinary coal oil lamp. For such practitioners as intend to make but occasional use of the laryngoscope, a reflector attached to a head band will answer very well, and will constitute all the accessory illuminating apparatus which it will be requisite to procure. Although, as stated, the light should be close to the head of the patient, and about on a level with his mouth, which affords us the most favorable conditions for examination ; it is possible to make a very satisfactory examination with the light at a distance from the patient, and considerably above his head, as when obtained from a gas-light, or reading lamp, in the patient's apartment. We must take care to have the light behind the patient. We select the most favorable spot to place him in, by trying the effect of the reflector upon the hand. When the patient is confined to bed, and it is impossible for him to sit up, he can be propped up with pillows, and an attendant can hold a light behind and above his head. Under these circumstances, the observer can use the head reflector to great advantage, inasmuch as he may have to lean over the bed or rest in great measure upon it.

In selecting a reflector, the focal distance should be measured by daylight, as the same mirror presents a much longer focus to artificial light, and we may thus have to place it at such a distance from the patient as to render the introduction of the laryngeal mirror uncomfortable to us.

### AUTO-LARYNGOSCOPY—SELF-EXAMINATION.

Every one who desires to master the subject of laryngoscopy should learn to examine his own larynx. This is most conveniently done by taking the patient's seat and holding a small toilet mirror before the eyes with one hand, while the laryngeal mirror is introduced with the other. Of course the autolaryngoscopist must be able to hold his tongue in the required position without extraneous assistance. The practice of autolaryngoscopy is not for the purpose of learning to manipulate upon others, for the movements required to introduce an instrument into one's own throat, are different from those employed in operating upon another. Its value consists in familiarizing us with the appearance of the laryngeal image, in enabling us to study for ourselves the effects of various normal and abnormal physiological effects, which we may learn to make use of in bringing to view parts which are not in the field of the mirror originally. Such movements are, variations in the performance of expiration and inspiration, intonation, vocalization, and cantation; the phenomena of sighing, coughing, retching; the retraction or elongation of the pharynx.

But to obtain skill in the examination of patients, the practitioner should employ every opportunity for so doing, as soon as he has mastered the regional anatomy of the parts. Practice alone makes perfect. The most rapid plan is for two or more individuals to study the art together, alternating for each other as patient and physician.

THE STRUCTURES THUS SUBJECTED TO OBSERVATION.

The obstacles to examination having been overcome, and the mirror introduced without difficulty and placed in proper position, it behooves us to consider what is likely to be reflected in the mirror, that we may be able to recognize the image. A little attention is necessary here, on the part of the beginner, because the method of examination is entirely different from that with which he is wont to examine other parts of the body. The dental mirror is the only instrument, constructed on the same principle, longer in use than the laryngoscope, and, in fact, it was the use of the dental mirror that suggested the idea of a laryngeal one. It must be remembered, then, that we are to see an image of the parts under examination, in the same way as we would look at the image of anything else in a looking-glass. We do not see the parts themselves. The structures reflected are seen in a reversed position, but retaining their relative relations with regard to right and left. The reader is again referred to fig. 4, for an appreciation of this appearance.

The structures most prominent in the mirror are, usually: the epiglottis, the cartilages of Santorini, and the vocal cords: there are readily recognized; but many other important parts are reflected, which are not easily located, until some familiarity has been acquired in laryngoscopic examination. It is the same as in the cultivation of observation under the microscope; many things are recognized, as we grow accustomed to the use of the microscope, which we did not see before; still they were in the field all the time.

The structures thus exposed to inspection are, from before, backwards: the posterior portion of the base of the tongue; the posterior surfaces of the anterior palatine arches, and their attachments to the sides of the base of the tongue; the lateral ligaments connecting the tongue with the hyoid bone; the ligaments connecting the tongue with the epiglottis, and the lingual sinuses between the middle glotto-epiglottic ligament, and the two lateral ligaments or folds; the tonsil glands; the posterior palatine arches, as they run downwards and backwards to be lost in the sides of the pharynx; the ligaments connecting the epiglottis and pharynx; the lateral ligaments connecting the epiglottis to the hyoid bone; the epiglottis itself, its anterior or lingual surface, its upper and lateral borders, and its posterior or laryngeal surface; the ligamentous folds of mucous membrane, connecting the sides of the epiglottis with the arytenoid cartilages, and forming the upper portion of the quadrangular membrane of the larynx; the arytenoid cartilages, with the cartilages of Santorini at their apex, imbedded in sub-mucous cellular tissue; the base of the cuneiform or Wrisberg cartilages, enclosed within the aryteno-epiglottic folds, a few lines from their arytenoidal extremities; the fold of mucous membrane between the arytenoidal cartilages; the pear-shaped sinuses just outside of the laryngeal cavity, comprising the space between the exterior surface of the quadrangular membrane of the larynx and the inner surface of the thyroid cartilage; the posterior wall of the pharynx, as far down as the position of the cricoid cartilage, where the entrance into the œsophagus appears as a narrow groove communicating with the pear-shaped or pyramidal sinuses; the upper cavity of the larynx and its component structures; the vocal cords, and above them the ventricles of Morgagni; the ventricular bands, known as false vocal cords, above the ventricles, and formed on each side by a duplicature of the laryngeal lining membrane; a portion of the lower cavity of the larynx, and the internal face of the anterior portion of the thyroid cartilage, crico-thyroid membrane, and cricoid cartilage; the anterior portion of the trachea, to a greater less extent, and under very favorable circumstances, clear down to the bifurcation of the tube; and, in exceptional cases, more or less of the continuity of the right bronchus; under peculiar circumstances, more or less of the posterior wall of the trachea.

Such are the structures which the laryngoscope enables us to examine in the living subject. It is impossible to bring all the tissues enumerated, into one and the same view; but by altering the direction of the mirror, a little forward or backward, to one side or the other, the light can be reflected upon all these structures in succession, and their condition observed. In moving the mirror, or giving it a further inclination to one side or the other, we must avoid any tremulousness. It is this constant hitting of the parts that induces irritable fauces. A decided, though gentle pressure upon the parts, is borne much better. It is for this reason, that the recommendation has already been made, to rest the lower edge of the mirror upon the posterior wall of the pharynx. In this way, we avoid titillation of the parts, which is so apt to ensue when we follow the rules laid down by several laryngoscopists, to avoid touching the soft palate, and particularly the pharynx. The only part to avoid, is the base of the tongue, contact with which induces retching. The handle of the mirror may be held in the centre of the mouth, or to one side, as is most convenient, or as the observer falls into the habit of doing.

## Figures and Tables

**Fig. 1. f1:**
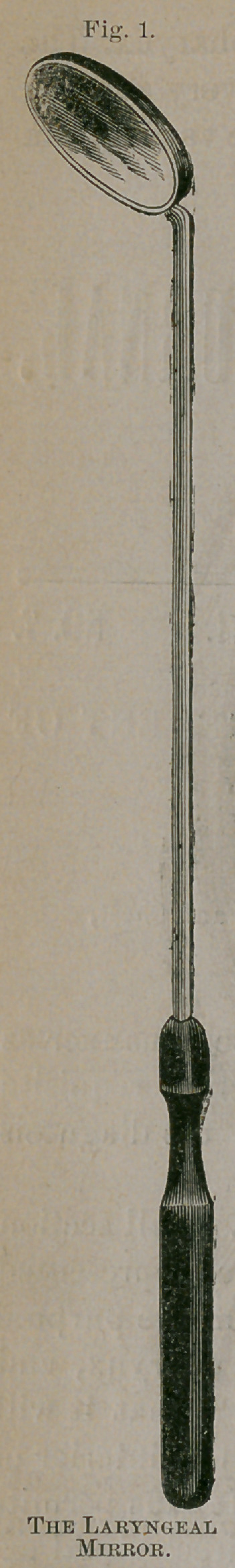


**Fig. 2. f2:**
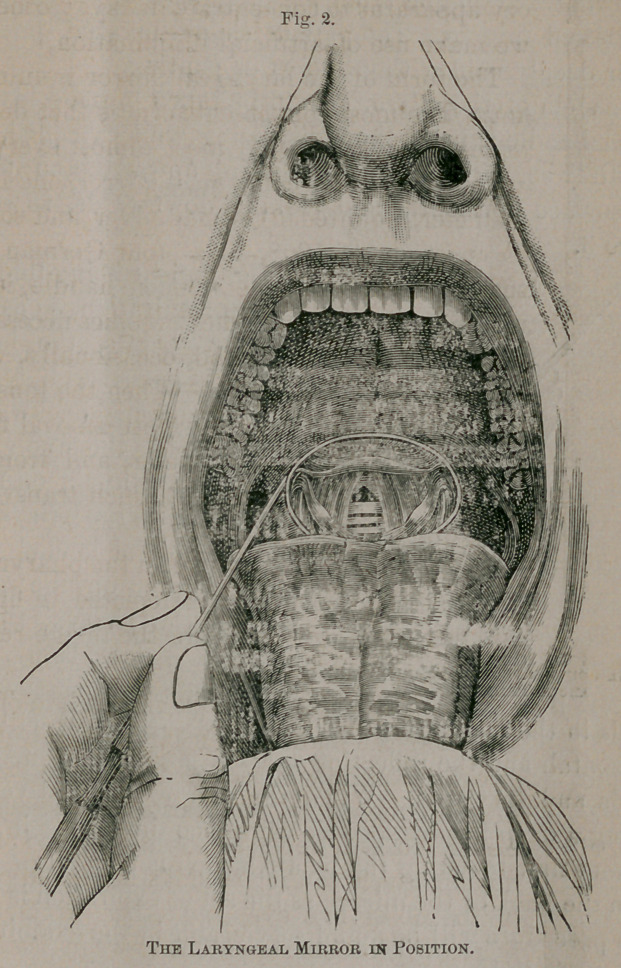


**Figure f3:**
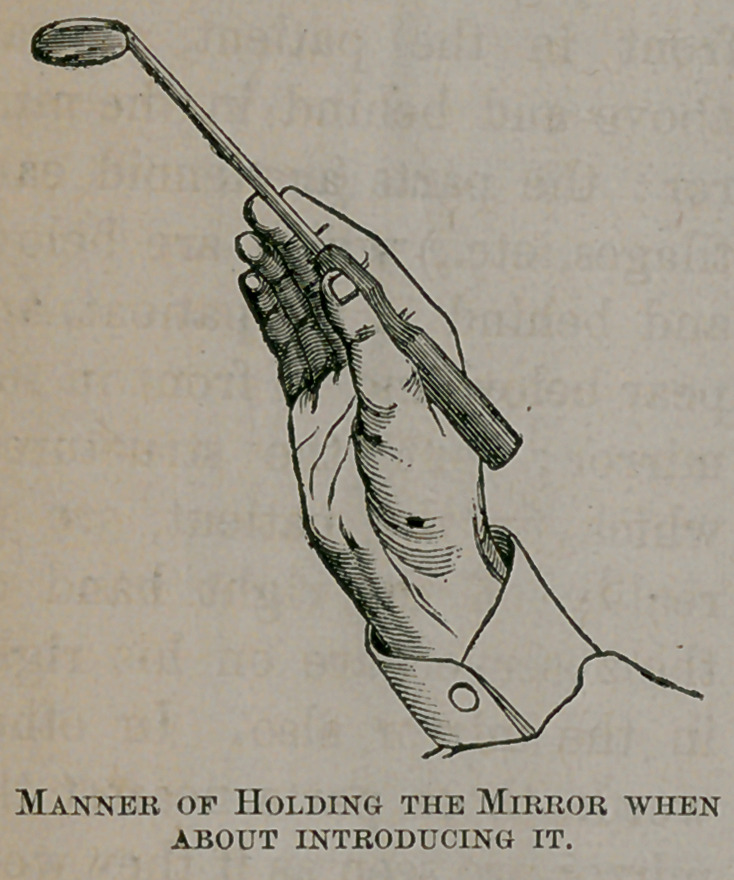


**Fig. 4. f4:**
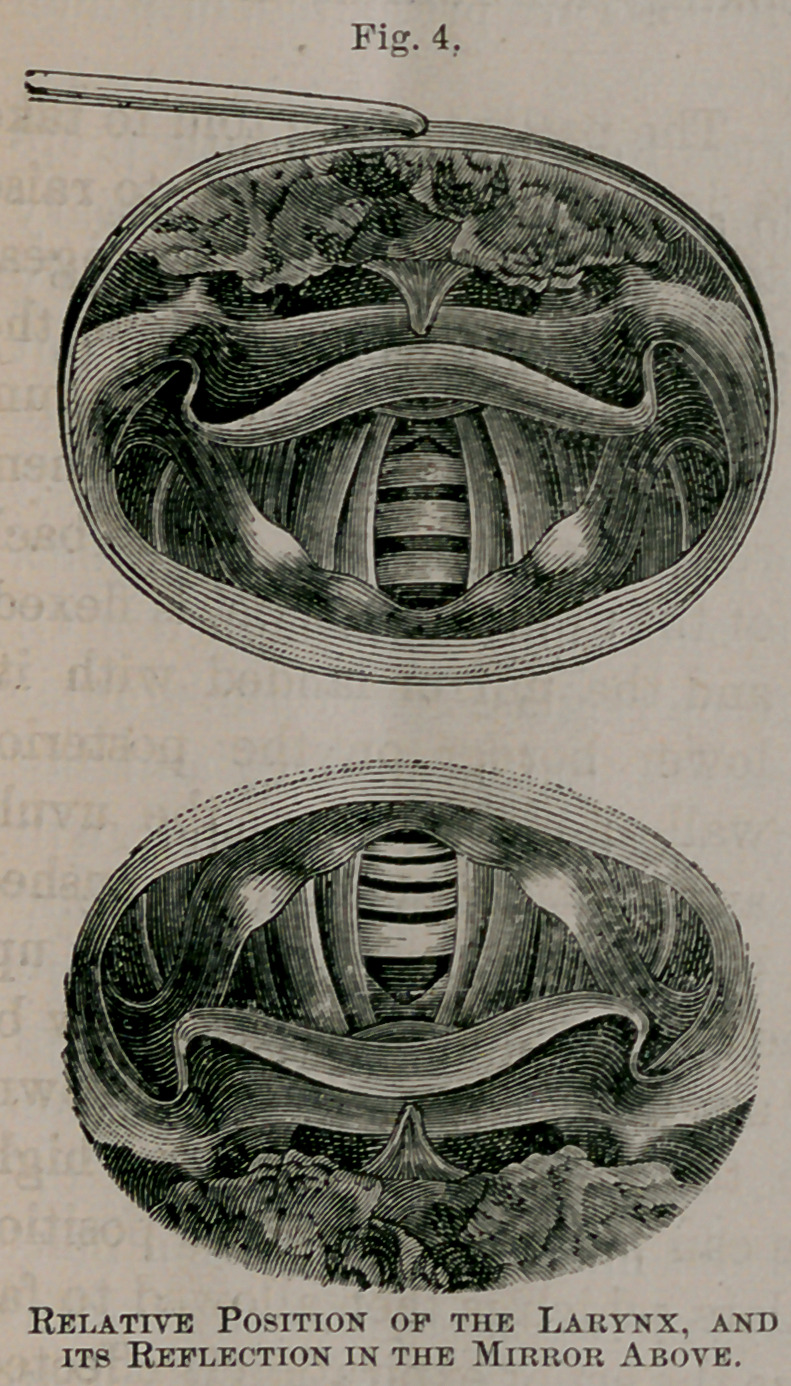


**Fig. 5. f5:**
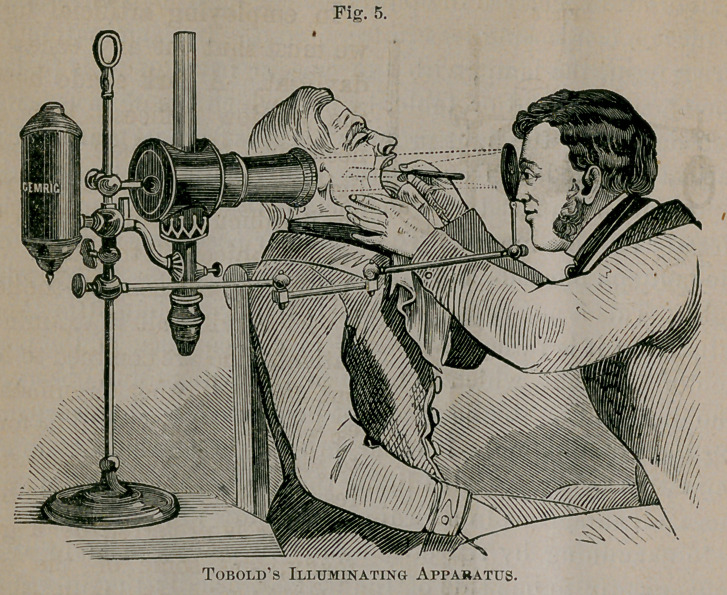


**Fig. 6. f6:**